# Detecting the presence of long-range temporal correlations in a time-varying measure of phase synchrony

**DOI:** 10.1186/1471-2202-12-S1-P104

**Published:** 2011-07-18

**Authors:** Maria Botcharova, Simon Farmer, Luc Berthouze

**Affiliations:** 1 CoMPLEX, Gower Street, University College London, WC1E 6BT, UK; 2 Institute of Neurology, University College London, London, WC1N 3BG, UK; 3 Centre for Computational Neuroscience and Robotics, University of Sussex, Falmer, BN1 9QH, UK; 4 Institute of Child Health, University College London, London, WC1N 1EH , UK

## 

Time-averaged coherence between human EEG and EMG signals has been found to increase with age of subject [1]. This is indicative of the underlying physiological system progressively reaching a level of synchrony, which allows it to perform tasks with efficiency. Recent experiments [2] have shown that physiological signals such as the EEG show long-range temporal correlations (LRTCs), implying that the brain may be poised in a critical state. It has been suggested that self-organization to such a critical state reflects a developing interaction of neuronal groups over a range of scales [3]. Since synchrony is an important method of communication between different neuronal groups it is of interest to determine whether its time course could reveal the presence of critical dynamics. To our knowledge, the only paper to have tackled this issue is [4], in which scale-free dynamics are suggested in fMRI and MEG data. This is done by demonstrating power laws in the probability distribution of phase-locked intervals between signals from related channels. Here, we present our own methodology, which relies on searching for LRTCs in a measure of instantaneous synchrony between two signals.

We first compute the analytical phases of two signals through the Hilbert Transform. The differential of the phase is taken to obtain a frequency measure, and the difference of these time series is calculated to provide an instantaneous synchrony level between the original signals. Detrended fluctuation analysis is then performed to ascertain the presence of LRTCs through an estimation of the Hurst exponent. LRTCs exist when the exponent is greater than 0.5 (a value of 0.5 indicates uncorrelated noise).

**Figure 1:**
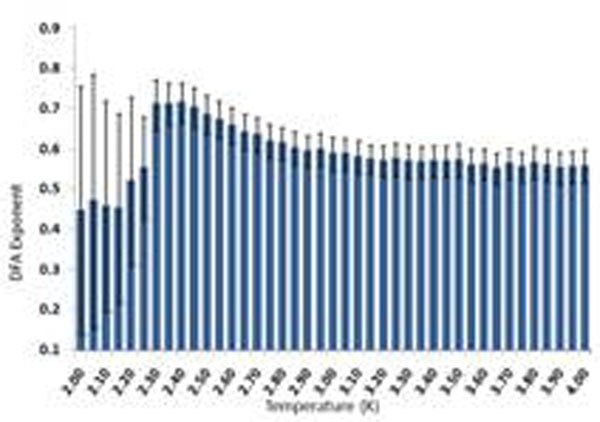
DFA exponents of synchrony between two regions in Ising model when the system undergoes transition from subcritical to supercritical state. The peak in DFA values is observed in the theoretically critical range of this system, 2.2-2.4. Error bars are standard deviation.

To demonstrate the method, we replicate the findings of [4] on the Ising model, which passes a transition from subcritical to supercritical behavior at a known parameter value. As shown in Figure 1, as the temperature in the Ising model is increased the system passes through a critical state (2.2-2.4) at which a non-trivial exponent is obtained (α=0.63). At non-critical temperatures, the exponent indicates a signal similar to white noise.

The methodology presented here displays greater generality than that described in [4]. Specifically, it avoids the use of an arbitrary threshold to determine the boundary of synchronization, and does not use time averaging in assessing instantaneous synchrony.

The construction of a time-varying phase synchrony measure between two signals and the determination of whether synchrony in a system is in a critical state has significant implications for understanding the development of the nervous system, when applied to neurophysiological data.
